# Arrhythmia and/or Cardiomyopathy Related to Maternal Autoantibodies: Descriptive Analysis of a Series of 16 Cases From a Single Center

**DOI:** 10.3389/fped.2019.00465

**Published:** 2019-11-20

**Authors:** Benzhen Wang, Sujuan Hu, Degong Shi, Zhen Bing, Zipu Li

**Affiliations:** ^1^School of Medicine, Shandong University, Jinan, China; ^2^Heart Center, Qingdao Women and Children's Hospital, Affiliated to Qingdao University, Qingdao, China; ^3^Department of Pediatrics, The Affiliated Hospital of Qingdao University, Qingdao, China; ^4^Department of Pediatrics, The Traditional Chinese Medical Hospital of Huangdao District, Qingdao, China

**Keywords:** autoantibody, congenital heart block, cardiomyopathy, steroid, neonatal lupus

## Abstract

**Objective:** To describe the clinical characteristics of maternal autoantibody-mediated arrhythmia and/or cardiomyopathy, and to explore the therapeutic role of glucocorticoids in these diseases.

**Methods:** This was a retrospective observational study of 2 fetuses and 14 children who presented with autoantibody-mediated arrhythmia and/or cardiomyopathy in our hospital from September 2010 to December 2018.

**Results:** In total, 16 patients were identified, including 2 fetuses, and 14 children. One mother suffered from Sjogren's syndrome, two suffered from systemic lupus erythematosus (SLE), and the remaining 13 were asymptomatic carriers of autoantibodies. Two fetuses were diagnosed with complete congenital heart block (CHB) and had mean heart rates of 45 and 50 bpm. In the 14 surviving children, third-degree CHB was detected in 4 children, second- to third-degree CHB in 4, corrected QT interval (QTc) prolongation in 1, atrioventricular dissociation, and junctional ectopic tachycardia in 1, complete left bundle branch block (CLBBB) with dilated cardiomyopathy (DCM) in 3, and endocardial fibroelastosis (EFE) in 1. All of the 14 surviving babies received intravenous immunoglobulin and glucocorticoids. None of the children received pacemaker implantation. During the follow-up, one 3-month-old girl who had complete CHB, DCM, and Torsades de pointes almost recovered after the administration of prednisone for ~8 years. Three cases with complete CHB had no improvement after 3–5 years of follow-up. One case with EFE and three cases with CLBBB and DCM were in stable condition now. Children with QTc prolongation and junctional ectopic tachycardia returned to a regular rhythm.

**Conclusions:** Autoantibody-mediated arrhythmias and/or cardiomyopathy are severe complications related to maternal autoantibodies, and the administration of steroid may be beneficial in reversing complete CHB.

## Introduction

Autoantibody-mediated heart disease, the most common manifestation of neonatal lupus, includes congenital heart block (CHB), and involvement beyond the atrioventricular node such as myocarditis, dilated cardiomyopathies (DCM), valvular abnormalities, and endocardial fibroelastosis (EFE) (1–3). Heart involvement of neonatal lupus carries an increased mortality and morbidity rate (4), and the mortality is between 16 and 23%, mostly *in utero* or during the first year of life (5).

Heart involvement in neonatal lupus is often accompanied by the presence of maternal autoantibodies in the fetal and neonatal circulation (6). The signature cardiac lesion is an atrioventricular block seen as a CHB, but 15–20% of these cases have associated fatal cardiomyopathy (7, 8). Autoimmune congenital heart block (ACHB), a rare condition that occurs in ~1 out of every 20,000 pregnancies (9), is associated with the transplacental passage of maternal autoantibodies such as anti-Ro/SSA and/or anti-La/SSB antibodies in more than 80% of affected neonates (3). ACHB might be detected *in utero* as a first- or second-degree atrioventricular block (AVB), but the majority have a potentially lethal complete AVB (CAVB) (10).

Atrioventricular block most commonly develops during the 18–24 weeks of gestation, and may be found using fetal Doppler echocardiography (11). The disease can continue to develop after birth, even during infancy and early childhood (12). At the same time, ~20% of affected fetuses can develop more diffuse myocardial disease manifested as cardiomyopathy and usually associated with endocardial fibroelastosis (EFE) (1, 2, 13); unfortunately, information relating to these conditions is sparse (2, 13, 14).

The majority of studies suggest that autoantibodies damage fetal conduction tissues leading to inflammation, calcification, and fibrosis, which can block signal conduction at the atrioventricular node without the requirement for additional structural abnormalities (2, 3). Indeed, increasing experimental, and clinical evidence has shown how these autoantibodies can critically interfere with cardiac electrical function and promote the development of life-threatening arrhythmic events by affecting the function of cardiac ion channels (7, 15).

The current curative CHB treatment is very controversial, and various therapeutic approaches including corticosteroids (especially fluorinated steroids, such as betamethasone or dexamethasone), intravenous immunoglobulin (IVIG), plasmapheresis, and beta-adrenergic agents have been reported (3, 5, 16). However, several studies have cast doubt on the efficacy of these therapies, and ultimately, a pacemaker is required in ~80% of newborns with congenital third-degree AVB (17–19).

This study aims to present our single-center experience describing the clinical characteristics of maternal autoantibody-mediated arrhythmia and/or cardiomyopathy. In addition, we describe one interesting case where the mother had systemic lupus erythematosus (SLE), and the obstetric history demonstrated fetal bradycardia, complete AVB, Torsades de pointes (Tdp), and DCM in the third month after birth, but had almost returned to normal following the long-term administration of corticosteroids.

## Materials and Methods

### Patient Identification

Clinical data on 16 cases were collected by performing a retrospective observational study of the patients at the Heart Center of Qingdao Women and Children's Hospital from September 2010 to December 2018. This study was approved by the ethics committee of Qingdao Women and Children's Hospital, and written informed consent was obtained from the parents of the study participants.

Inclusion criteria were as follows: The presence of maternal autoantibodies, such as anti-Ro/SSA and/or anti-La/SSB antibodies; and the confirmation of arrhythmia [including second- and third-degree heart block, prolonged QT interval, and complete left bundle branch block (CLBBB)] and/or cardiomyopathy (autoantibody-mediated EFE or DCM) in the patients, as documented by fetal echocardiography, electrocardiogram, or Holter monitoring performed in our center, and excluding other causes, such as myocarditis, complications from heart surgery, inherited cardiomyopathy, trauma, or toxication (1, 13, 20–22).

### Selected Variables for Analysis

For the 16 mothers included in this study, demographic characteristics such as the age at diagnosis of CHB and type of autoimmune disease, and immunologic features such as the presence of anti-nuclear antibody, anti-Ro/SSA, anti-Ro/SSB, anti-Ro52, and anti-Ro 60 were collected when the children's diseases were first diagnosed. A screening of autoimmune diseases was also performed in asymptomatic carriers of autoantibodies.

Among the 16 fetuses and children, the gestation age at CHB diagnosis, age at CHB diagnosis, and type of CHB were collected. In addition, among the surviving children, we collected data on the sex, birth weight, heart rate, heart malformation, type of cardiomyopathy, clinical manifestation, presence of autoantibodies, erythrocyte sedimentation rate (ESR), size of ventricular cavity, heart function, treatment, and the follow-up.

### Statistical Methods

The results from continuous variables are presented as median (range), while those from categorical data are presented as percentages.

## Results

A total of 16 women, accounting for 2 fetuses and 14 children with arrhythmia and/or cardiomyopathy, were identified from the cohort of the obstetrics clinic and the department of pediatrics in our hospital (Table 1). Termination of pregnancy (TOP) was performed in two women because of the persistence of complete CHB, and 14 live-born infants were delivered at full-term.

**Table 1:** Features of mothers, fetuses, and children.

**Maternal features**	
Mean age (years)	30 (27–35)
**Systemic autoimmune disease**	
Sjogren's syndrome	1 (6.2%)
Systemic lupus erythematosus	2 (12.5%)
Asymptomatic carrier	13 (81.3%)
**Autoantibodies**	
ANA + Anti-Ro/SSA	3 (18.8%)
ANA + Anti-Ro/SSB	1 (6.2%)
ANA + Anti-Ro/SSA + Anti-Ro/SSB + Anti-Ro/52	3 (18.8%)
ANA + Anti-Ro/SSA + Anti-Ro/52	5 (31.2%)
ANA + Anti-Ro/SSA + Anti-Ro/52 + Anti-Scl-70	1 (6.2%)
ANA + Anti-Ro/SSA + Anti-Ro/52 + Anti-nucleosome antibody	1 (6.2%)
Anti-Ro/52 + Anti-PM-SCL antibody	1 (6.2%)
Anti-Ro/SSA + Anti-Ro/52	1 (6.2%)
**Features of fetuses and children**	
Gestation age at the diagnosis (weeks, 2 fetuses)	28, 36
Mean age at diagnosis after birth (months, 14 surviving children)	14 (3–108)
**Sex**	
Female	10 (71.4%)
Male	4 (28.6%)
NA	2 (12.5%)
**Clinical manifestation of surviving children**	
Retardation	6 (42.9%)
Seizure	1 (7.1%)
None	7 (50%)
**Type of Arrhythmia of surviving children**	
Third-degree AVB	4 (28.5%)
Second → third-degree AVB	4 (28.5%)
CLBBB	3 (21.4%)
AV dissociation + functional ectopic tachycardia	1 (6.2%)
QTc prolongation	1 (6.2%)
None	1 (6.2%)
**Type of cardiomyopathy of Surviving children**	
EFE	1 (7.1%)
DCM	3 (21.4%)
None	10 (71.4%)
**Autoantibodies of surviving children**	
Anti-nuclear antibody	2 (14.3%)
Anti-Ro/52	2 (14.3%)
DS-DNA	1 (7.1%)
Anti-PM-SCL	1 (7.1%)
None	8 (57.1%)

*NA, not applicable; ANA, antinuclear antibodies; AVB, atrial ventricular block; AV, atrial ventricular; CLBBB, complete left bundle branch block; DS-DNA, double-stranded deoxyribonucleic acid; EFE, endocardial fibroelastosis; DCM, dilated cardiomyopathies*.

Unfortunately, data to calculate the incidence of maternal autoantibody-mediated arrhythmia and/or cardiomyopathy in the heart center (from 2010 to 2018) are not available.

### Maternal Features

The traits of the women whose children were diagnosed with arrhythmia and/or cardiomyopathy are shown in Table 1. The median age at diagnosis of autoimmune abnormality was 30 years (range, 27–35 years). Three of the 16 women suffered from autoimmune disease, of whom one (6.2%) had Sjogren's syndrome and two (12.5%) had SLE. However, the remaining 13 women were asymptomatic carriers of autoantibodies. All of the mothers had positive autoantibodies, of whom 14 (85.7%) had positive anti-nuclear antibody (ANA), 13 (78.6%) had positive anti-Ro/SSA, 11 (64.3%) had positive anti-Ro52, 4 (25%) had positive anti-Ro/SSB, and 3 (18.8%) had positive anti-Ro 60. Only one case (7.7%) had positive anti-nucleosome antibodies, anti-scl-70, and anti-PM-Scl. Both mothers of fetus had positive anti-Ro/SSA and anti-Ro52, and erythrocyte sedimentation rate (ESR) of one of them reached 80 mm/h. The cases with coexisting antibodies are shown in Table 1.

### Features of Children and Fetuses

The main characteristics of the children and fetuses are shown in Tables 1, 2. The gestational age at the diagnosis of complete CHB in the two aborted fetuses was 28 and 30 weeks, and the mean heart rate at diagnosis of CHB was 45 and 50 bpm. In the 14 surviving children, 12 (85.7%) were full-term infants and 2 were premature infants. Ten cases were female and four cases were male. The median birth weight was 3,000 g (range, 2,000–3,700 g), and the median age at diagnosis was 14 months (range, 3–108 months). The data confirmed third-degree AVB in four babies, second- to third-degree AVB in four, QTc prolongation in one, junctional ectopic tachycardia in one, EFE in one, and CLBBB combined with DCM in three (Table 1). Case 4 was a special case who manifested with third-degree AVB, Tdp, and DCM; this case is described in detail below.

**Table 2:** Clinical characteristics of 16 cases with autoantibody-mediated heart disease.

**Case**	**Age at diagnosis (gestational age)**	**Delivery**	**Sex**	**Birth weight (g)**	**Type of disease (degree of AVB)**	**FHR (beats/min)**	**NT-proBNP (ng/ml)**	**LVEDD (mm)**	**LVEF (%)**	**Treatment**	**Outcome**
1	19 months	Full-Term	F	3700	3	NA	218.7	34	N	IVIG + GC + SAL + ATR	Stay
2	4 months	Full-Term	F	3600	3	NA	624	N	N	IVIG + GC + SAL + ATR	Stay
3	14 months	Full-Term	F	3400	2 → 3 + ASD	98	323	N	N	IVIG + GC + SAL + ATR + Surgery	Improve
4	3 months	Full-Term	F	3100	3 + Tdp + DCM	80	6000	35	33	IVIG + GC + SAL + CAP	Improve (nearly cured)
5	60 months	Full-Term	F	3200	3 + PDA	55	103	35	N	IVIG + GC + SAL + ATR+ Intervention	Stay
6	32 weeks	TOP	NA	NA	3	40	NA	NA	NA	NA	NA
7	108 months	Full-Term	F	3300	2 → 3	N	63	N	N	IVIG + GC + SAL	Stay
8	6 months	Full-Term	F	2800	2 → 3 + ASD	N	NA	N	N	IVIG + GC + SAL+ Intervention	Improve
9	39 months	Full-Term	F	3000	2 → 3 + PDA	N	248	N	N	IVIG + GC + SAL + CAP	Stay
10	3 months	Full-Term	F	3000	CLBBB + DCM	N	2000	39	30	IVIG + GC + SAL + CAP + D	Improve
11	5 months	Full-Term	M	2200	CLBBB + DCM	N	> 35000	56	24	IVIG + GC + SAL + CAP + D	Improve
12	56 months	Full-Term	F	3000	CLBBB + DCM	N	2000	58	31	IVIG + GC + SAL + CAP + D	Stay
13	3 months	Full-Term	M	3550	EFE	N	> 35000	39	20	IVIG + GC + SAL + CAP + D	Improve
14	36 weeks	Pre-Term	M	3000	AV dissociation + JT	200	8613	N	N	IVIG + GC + AMI	Improve
15	28 weeks	TOP	NA	NA	3	50	NA	NA	NA	NA	NA
16	34 weeks	Pre-Term	M	2000	QTc prolongation	70	14774	N	N	IVIG + GC + SAL	Recover

*N, normal; NA, not applicable; FHR, fetal heart rate; NT-proBNP, N-terminal pro B-type natriuretic peptide; LVEDD, left ventricular end-diastolic dimension; LVEF, left ventricular ejection fraction; AVB, atrioventricular block; AV, atrioventricular; Tdp, Torsades de pointes; TOP, termination of pregnancy; EFE, endocardial fibroelastosis; DCM, dilated cardiomyopathy; F, female; M, male; ASD, atrial septal defect; PDA, patent ductus arteriosus; CLBBB, complete left bundle branch block; JT, junctional tachycardia; IVIG, intravenous immunoglobulin; GC, glucocorticoid; SAL, salbutamol; CAP, captopril; AMI, amiodarone; D, diuretic; ATR, atropine*.

Among the 14 survivors, convulsions were the initial symptom in one case, and development retardation was the most common symptom in six children. The mean heart rates were below 100 bpm, and the median heart rate was 77 bpm (range, 50–99 bpm) in seven children. Four of the 14 surviving babies were diagnosed with congenital heart disease, including secundum atrial septal defect (ASD) (the sizes of the defects were 8 mm × 7 mm and 13 mm × 15 mm) and patent ductus arteriosus (PDA) (the ductal diameters were 3 and 2 mm) in two children. An echocardiogram was also used to assess ventricular size and ejection fraction (EF), and 8 of the 14 babies had an enlarged left ventricle, with a left ventricular end-diastolic dimension (LVEDD) of between 34 and 58 mm, and of whom five babies had left ventricular systolic dysfunction with a left ventricular ejection fraction (LVEF) below 40%.

The 14 surviving babies were all tested for autoantibodies, and the results showed that two babies were positive for ANA coexisting with anti-Ro/SSA, and double-stranded deoxyribonucleic acid (DS-DNA) and anti-PM-SCL antibodies were positive in one baby. However, the other babies had no significant level of autoantibodies. Furthermore, ESR and thyroid function were normal in all of the surviving babies (Table 1).

All of the surviving babies received IVIG and glucocorticoid (methylprednisolone and/or prednisolone) treatment. The IVIG was applied five times in total. The first dose of IVIG was 2 g/kg, then 1 g/kg was applied after 2 weeks, and then 1 g/kg per time was applied every other month for three times. Methylprednisolone is generally selected as glucocorticoid (prednisolone approximate equivalent dose conversion with methylprednisolone is 4:5), 2 mg/kg for 1 week, then reduced to 1 mg/kg for 1 month, and then reduced to 0.5 mg/kg for 2–6 months (the duration of application is adjusted according to the condition of the disease; poor response to treatment led us to suspend the drug, and conversely, the treatment will be maintained). Five children with third-degree CHB were administrated salbutamol and atropine, and those with cardiac dysfunction were given diuretics, captopril, and symptomatic treatment and one boy with EFE also received digoxin. None of the babies underwent pacemaker implantation; one child had surgical repair of ASD, and two children had transcatheter closure of ASD or PDA. At the follow-up, no improvement was found in four of the five cases with third-degree CHB, and one case with EFE and three cases with CLBBB combined with DCM were in a stable condition with the exception of growth retardation; however, no significant improvement in the enlarged left ventricle was noticed. Two newborns with QTc prolongation or atrioventricular dissociation recovered to a regular rhythm after the administration of IVIG and glucocorticoids (Table 2).

**Figure 1:**
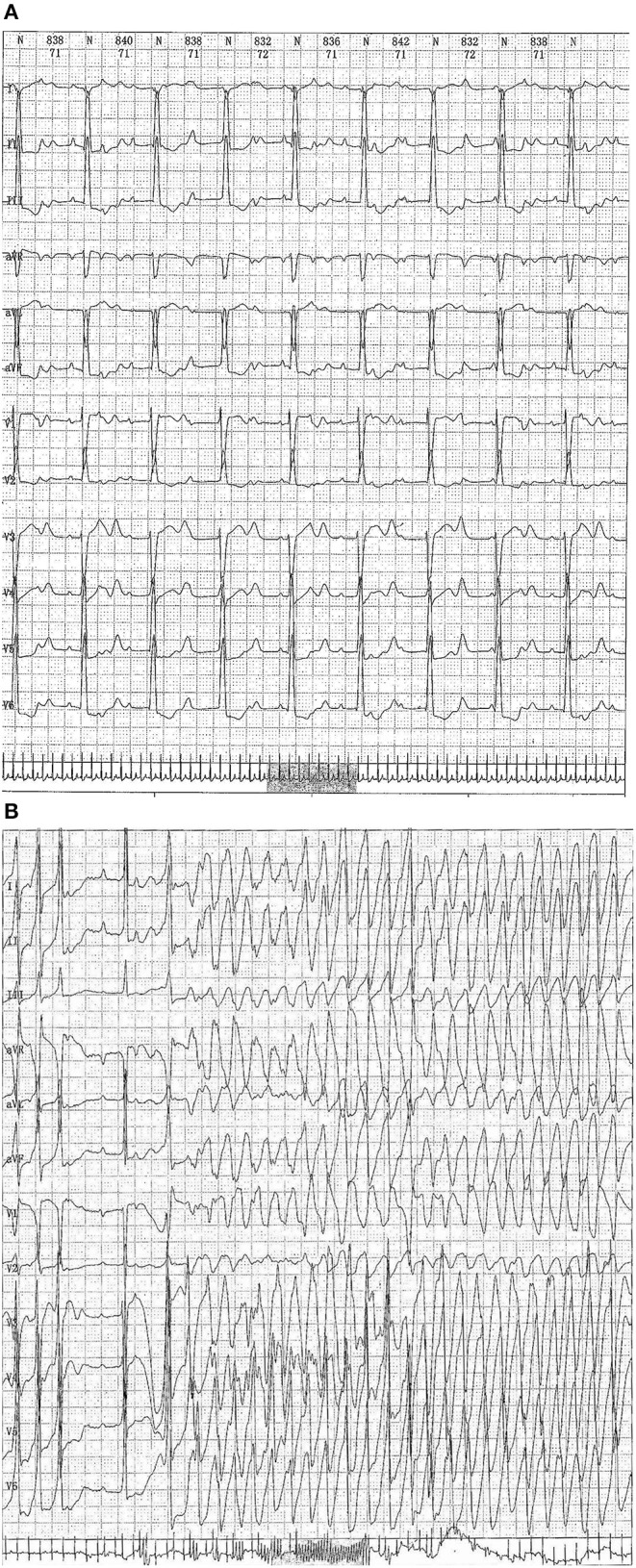
**(A)** Echocardiograph showing a third-degree atrioventricular block. **(B)** Electrocardiography showing a third-degree atrioventricular block combined with Torsades de pointes (Tdp) (up to a maximum duration of 68 s).

**Figure 2:**
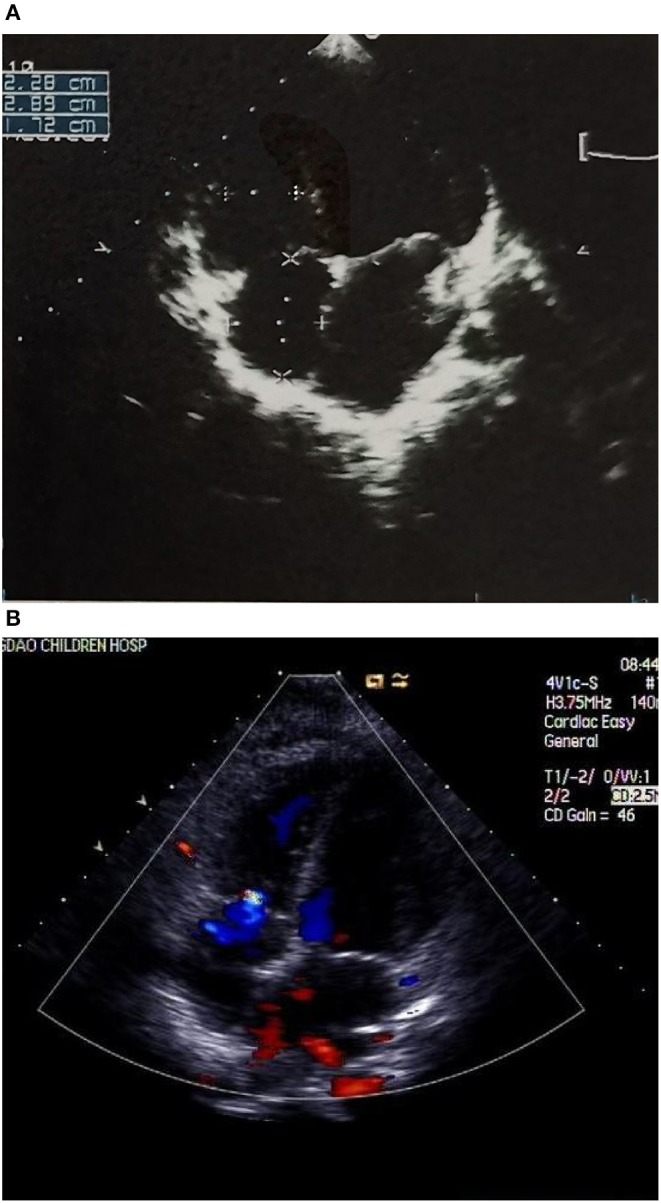
**(A)** Echocardiograph showing an enlarged left ventricle (LVEDD 35 mm) **(B)** Echocardiograph showing a relatively normal heart size (LVEDD 45 mm) and a mild tricuspid regurgitation.

**Figure 3:**
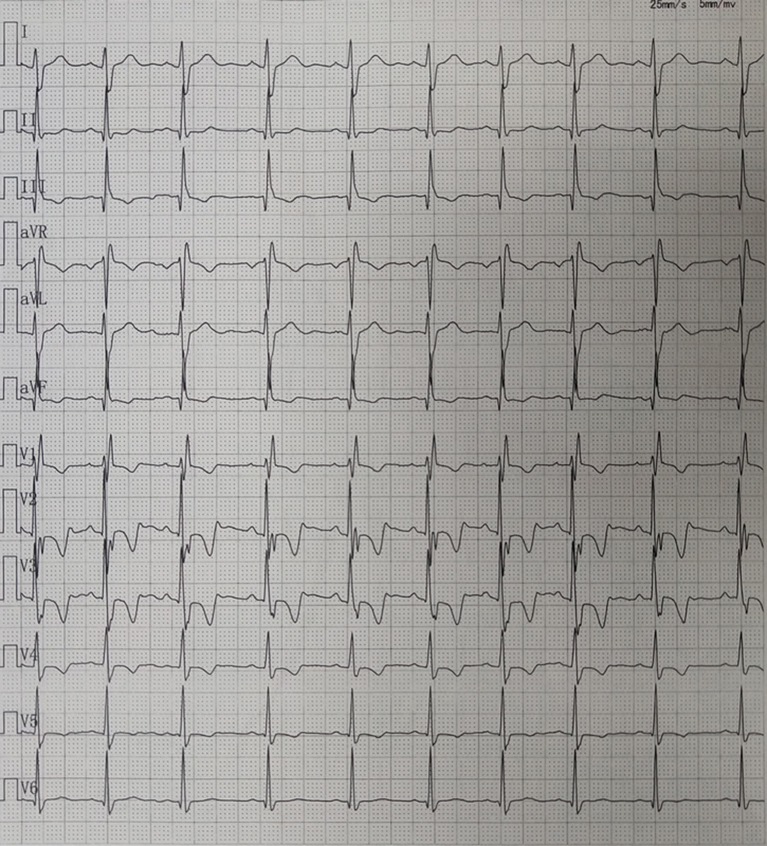
Electrocardiography showing sinus rhythm and complete right bundle branch block (CRBBB) with abnormal Q wave on leads II, III, avF, and V6.

Case 4 was an interesting case; she had convulsion as the initial clinical manifestation in the third month after birth, and was diagnosed with third-degree AVB (Figure 1A), Tdp (Figure 1B), and DCM, and the obstetric history of her mother revealed fetal bradycardia during labor; unfortunately, we did not obtain the result of the fetal heart ultrasound. Her mother was diagnosed with SLE because a laboratory investigation showed an ESR of 80 mm/h, an ANA titer of 1:1,000, and positive anti-Ro/SSA and anti-Ro/52 antibodies; however, no autoantibodies were found in the baby. An electrocardiogram showed that the baby had a ventricular rate of 60 bpm and an atrial rate of 140 bpm, paroxysmal ventricular tachycardia, and TdP. An echocardiogram showed normal cardiac anatomy with an enlarged left ventricle (LVEDD 35 mm) (Figure 2A), diminished contractility (LVEF 42%), moderate mitral regurgitation, and mild pulmonary hypertension with a pulmonary artery systolic pressure of 33 mmHg. The baby was treated using steroids (prednisone), intravenous immunoglobulin, salbutamol, atropine, diuretic (furosemide), and captopril, but no permanent pacemaker was implanted. During her 8-year clinic visit, the electrocardiogram and echocardiogram showed an improvement in heart rhythm and function, and the dosage of prednisone was subsequently reduced from 2 mg/kg/day to 0.5 mg/kg once every other day step-by-step. At the same time, salbutamol, and atropine were gradually decreased, and was ceased at the relief of symptoms. Finally, her third-degree AVB completely disappeared, no further ventricular tachycardia or Tdp was observed, her heart rhythm restored to sinus rhythm, and her mean heart rate increased to 75 bpm. Only the complete right bundle branch block (CRBBB) with abnormal Q wave on leads II, III, avF, and V6 remained (Figure 3). On the other hand, an echocardiogram revealed a relatively normal heart size (LVEDD 45 mm) and function (LVEF 63%) (Figure 2B).

## Discussion

In this study, we describe the clinical and immunological characteristics of maternal autoantibody-mediated arrhythmia and/or cardiomyopathy in “children” rather than neonates. Combined with the children's electrocardiogram and echocardiographic features and significant abnormalities of the maternal immune system, we considered whether the diagnosis of autoantibody-mediated arrhythmia and/or cardiomyopathy was appropriate for the children included in our study. This was considered even in cases where we missed the optimal time to confirm the transplacental passage of maternal autoantibodies. Interestingly, in China, a delay in the management of high-grade AVB or other arrhythmias in the perinatal period is very common. There are several possible reasons for this situation. First, the testing for autoantibodies is not routinely performed during antenatal care. Second, techniques such as fetal Doppler echocardiography are not being performed effectively and screening for cardiovascular disease is often the weak link in newborn screening. Our report is consistent with the previous study that demonstrated that the majority of autoantibody-positive mothers who deliver a child with ACHB are asymptomatic for autoimmune disease (3). This situation increases the difficulty for prenatal diagnosis of autoantibody-mediated disease. Three mothers in our series were positive for anti-Scl-70, anti-nucleosome antibody, and anti-PM-SCL antibody; however, no previous studies have focused on the relationship between these antibodies and autoantibody-mediated arrhythmia or cardiomyopathy.

Autoimmune cardiac channelopathies are a novel and increasingly recognized mechanism of cardiac arrhythmias and/or cardiomyopathy; these are mediated by circulating autoantibodies that interfere with the function of various cardiac ion channels (15). Indeed, autoantibodies critically interfere with cardiac electrical function and promote the development of life-threatening arrhythmic events by affecting the function of cardiac ion channels (15). A large number of studies have clearly demonstrated that anti-Ro/SSA autoantibody cross-reactivity with L-type Ca^2+^ channels, and subsequent inhibition of I_CaL_ in the fetal heart conduction system are crucial pathogenic mechanisms through which these autoantibodies lead to the development of ACHB (15, 23–25). Increasing evidence indicates that the hERG1 K^+^ channel is also a specific target of anti-Ro/SSA, which constitutes a novel form of acquired long QT syndrome (LQTS) of autoimmune origin (15, 26). Moreover, there are several other autoantibodies capable of disturbing various ion channels such as the anti-T-type Ca^2+^ channel, anti-KCNQ1 K^+^ channel, and the anti-Na^+^ channel; all of the above antibodies might lead to arrhythmia and/or cardiomyopathy (15). In terms of maternal demographic and clinical data, our results are generally consistent with the majority of previous studies in that the vast majority of the mothers had anti-Ro/SSA antibodies and four mothers also had anti-La/SSB antibodies.

Dilated cardiomyopathies can be diagnosed *in utero* together with autoimmune CHB, or may develop after birth; when DCM develops after the neonatal period, it is referred to as late-onset or delayed DCM (20, 22, 27). Morel et al. found that 22 of 174 (12.7%) children with CHB had no signs of DCM, either at birth or during the neonatal period, but ultimately developed late-onset DCM during the follow-up. However, neonatal DCM was observed in 13 children with CHB (6.9%) (20). It is noteworthy that three (21.4%) children had CLBBB complicated with DCM in our series. To date, there are no data regarding the incidence of new-onset LBBB in young patients with DCM; generally, the conduction abnormalities are a part of the natural history of DCM (28). The previous study has shown that myocardial fibrosis correlates with CLBBB (29). Based on the available evidence, it is difficult to determine whether DCM or CLBBB was the initial problem for these three babies. In addition, EFE also occupies a certain proportion of the cardiac manifestation of neonatal lupus (9, 20) and isolated EFE associated with maternal antibodies can be present in the absence of CAVB (13). In our study, a 3-month-old boy whose mother had Sjogren's syndrome was diagnosed with EFE without conduction abnormalities; fortunately, the IVIG and glucocorticoid achieved some positive clinical effects. Although information relating to DCM or EFE caused by autoantibodies remains sparse, we believe that the incidence of this condition induced by autoantibodies is relatively common in clinical practice. Therefore, further research is needed to explore the relationship between cardiomyopathy and autoantibodies, especially maternal autoantibodies.

The therapeutic principle is extremely similar for heart block and other heart involvements of neonatal lupus, including corticosteroids, and intravenous immunoglobulin. Additionally, beta-adrenergic agents, and pacemaker implantation are particularly crucial for patients with third-degree AVB and an extremely low heart rate (1, 3, 5, 6, 12, 18, 19). To date, a series of studies have shown that the IVIG might be effective in preventing the passively acquired autoimmune CHB by several potential mechanisms: First, by lowering or even eliminating maternal antibodies in the fetal circulation; then, by decreasing the placental transport of maternal antibodies; and finally, by regulating the fetal and neonatal immune system (6, 17–19). Although not uniformly effective, corticosteroids, especially fluorinated steroids, have been associated with the reversal of first- and second-degree heart block; however, third-degree heart block has not yet been reversed with any treatment (1, 5, 19). Furthermore, considering the side effects of steroids, the routine administration of steroids remains controversial.

To the best of our knowledge, case 4 in our study is the first case of CAVB, Tdp, and DCM that has been almost cured by the long-term administration of glucocorticoid; this case also provides us with some insight into the effect of steroids. The 3-month-old girl had third-degree heart block, Tdp, and DCM; after a relatively long duration of prednisone treatment, the heart block, severe ventricular tachycardia, as well as the heart size and function gradually restored to almost normal. So far, the growth and development of this girl are similar to that of her peer group and no obvious side effects have been observed. This therapeutic effect reflects the immune-mediated nature of this disease and suggests that steroids might actually have the capacity to reverse complete heart block. More importantly, this is the newest and most remarkable evidence for a possibility that complete heart block may be recoverable rather than irreversible. Although this opinion is contrary to all previous research (1, 5, 19), it is an exciting area that should be investigated further. Despite the success of this case, this approach also has great risks and is not recommended as a regular treatment in clinical practice without ample evidence.

Our study has a number of limitations owing to its retrospective design, the rarity of autoantibody-mediated arrhythmia, and/or cardiomyopathy and the lack of *in utero* details, which limit the power of the analyses. Furthermore, we were unable to determine the exact date when the heart involvement occurred. Ultimately, since only one child had a significant improvement with the usage of steroids, we could not evaluate the underlying mechanism and full effect of this therapy. Thus, more research, both experimental and clinical, should be conducted in order to fully elucidate these points.

In conclusion, autoantibody-mediated arrhythmias and/or cardiomyopathy are severe complications related to maternal autoantibodies, mainly anti-Ro/SSA and anti-La/SSB antibodies. Although the administration of steroids may have the ability to reverse complete AVB, further studies are needed to understand the mechanisms and to determine the security and validity of these findings.

## Data Availability Statement

All datasets generated for this study are included in the article/supplementary material.

## Ethics Statement

The studies involving human participants were reviewed and approved by the ethics committee of Qingdao Women and Children's Hospital. Written informed consent to participate in this study was provided by the participants' legal guardian/next of kin. Written informed consent was obtained from the individual(s), and minor(s)' legal guardian/next of kin, for the publication of any potentially identifiable images or data included in this article.

## Author Contributions

BW was responsible for interpretation of the data, statistical analysis, drafting of the manuscript, and approval of the final version to be published. SH, DS, ZB, and ZL were responsible for the study conception, data collection and interpretation, revision of the manuscript, and approval of the final version to be published. All authors read and approved the final manuscript.

### Conflict of Interest

The authors declare that the research was conducted in the absence of any commercial or financial relationships that could be construed as a potential conflict of interest.

## References

[1] Trucco SMM Jaeggi EM Cuneo BM Moon-Grady AJM Silverman EM Silverman NM. Use of intravenous gamma globulin and corticosteroids in the treatment of maternal autoantibody-mediated cardiomyopathy. JACC. (2011) 57:715–23. 10.1016/j.jacc.2010.09.044 21292131

[2] Nield LE Silverman ED Taylor GP Smallhorn JF Mullen JB Silverman NH. Maternal anti-Ro and anti-La antibody-associated endocardial fibroelastosis. Circulation. (2002) 105:843–8. 10.1161/hc0702.104182 11854125

[3] Brito-Zeron P Izmirly PM Ramos-Casals M Buyon JP Khamashta MA. The clinical spectrum of autoimmune congenital heart block. Nat Rev Rheumatol. (2015) 11:301–12. 10.1038/nrrheum.2015.29 25800217 PMC5551504

[4] Tincani A Rebaioli CB Taglietti M Shoenfeld Y. Heart involvement in systemic lupus erythematosus, anti-phospholipid syndrome and neonatal lupus. Rheumatology. (2006) 45(Suppl 4): iv8–iv13. 10.1093/rheumatology/kel308 16980725

[5] Brucato A Tincani A Fredi M Breda S Ramoni V Morel N. Should we treat congenital heart block with fluorinated corticosteroids? Autoimmun Rev. (2017) 16: 1115–8. 10.1016/j.autrev.2017.09.005 28899797

[6] Buyon JP Clancy RM Friedman DM. Cardiac manifestations of neonatal lupus erythematosus: guidelines to management, integrating clues from the bench and bedside. Nat Clin Pract Rheumatol. (2009) 5:139–48. 10.1038/ncprheum1018 19252519

[7] Lee HC Huang KT Wang XL Shen WK. Autoantibodies and cardiac arrhythmias. Heartrhythm. (2011) 8:1788–95. 10.1016/j.hrthm.2011.06.032 21740882 PMC3855646

[8] Moak JP Barron KS Hougen TJ Wiles HB Balaji S Sreeram N. Congenital heart block: development of late-onset cardiomyopathy, a previously underappreciated sequela. JACC. (2001) 37:238–42. 10.1016/S0735-1097 11153745

[9] Buyon JP Clancy RM. Neonatal lupus: basic research and clinical perspectives. Rheum Dis Clin North Am. (2005) 31:299–313. 10.1016/j.rdc.2005.01.010 15922147

[10] Strasburger JF Wakai RT. Fetal cardiac arrhythmia detection and in utero therapy. Nat Rev Cardiol. (2010) 7:277–90. 10.1038/nrcardio.2010.32 20418904 PMC2995324

[11] Buyon JP Hiebert R Copel J Craft J Friedman D Katholi M. Autoimmune-associated congenital heart block: demographics, mortality, morbidity and recurrence rates obtained from a national neonatal lupus registry. JACC. (1998) 31:1658–66. 10.1016/S0735-1097 9626848

[12] Ruffatti A Marson P Svaluto-Moreolo G Marozio L Tibaldi M Favaro M. A combination therapy protocol of plasmapheresis, intravenous immunoglobulins and betamethasone to treat anti-Ro/La-related congenital atrioventricular block. A case series and review of the literature. Autoimmun Rev. (2013) 12:768–73. 10.1016/j.autrev.2013.01.002 23340276

[13] Nield LE Silverman ED Smallhorn JF Taylor GP Mullen JB Benson LN. Endocardial fibroelastosis associated with maternal anti-Ro and anti-La antibodies in the absence of atrioventricular block. JACC. (2002) 40:796–802. 10.1016/S0735-1097 12204513

[14] Nield LE Silverman ED Taylor GP Maternal anti-Ro and anti-La antibody associated endocardial fibroelastosis. Acccurr J Rev. (2002) 11:103 10.1016/S1062-1458(02)00756-0 11854125

[15] Lazzerini PE Capecchi PL Laghi-Pasini F Boutjdir M. Autoimmune channelopathies as a novel mechanism in cardiac arrhythmias. Nat Rev Cardiol. (2017) 14:521–35. 10.1038/nrcardio.2017.61 28470179

[16] Gleicher N Elkayam U. Preventing congenital neonatal heart block in offspring of mothers with anti-SSA/Ro and SSB/La antibodies: a review of published literature and registered clinical trials. Autoimmun Rev. (2013) 12:1039–45. 10.1016/j.autrev.2013.04.006 23684701

[17] Friedman DM Llanos C Izmirly PM Brock B Byron J Copel J. Evaluation of fetuses in a study of intravenous immunoglobulin as preventive therapy for congenital heart block: Results of a multicenter, prospective, open-label clinical trial. Arthritis Rheum. (2010) 62:1138–46. 10.1002/art.27308 20391423 PMC3214993

[18] Friedman DM Kim MY Copel JA Llanos C Davis C Buyon JP. Prospective evaluation of fetuses with autoimmune-associated congenital heart block followed in the PR Interval and Dexamethasone Evaluation (PRIDE) Study. Am J Cardiol. (2009) 103:1102–6. 10.1016/j.amjcard.2008.12.027 19361597 PMC2730772

[19] Ruffatti A Cerutti A Favaro M Del RT Calligaro A Hoxha A Marson P. Plasmapheresis, intravenous immunoglobulins and bethametasone - a combined protocol to treat autoimmune congenital heart block: a prospective cohort study. Clin Exp Rheumatol. (2016) 34:706–13. Available online at: https://www.clinexprheumatol.org/abstract.asp?a=10107 27385463

[20] Morel N Lévesque K Maltret A Baron G Hamidou M Orquevaux P. Incidence, risk factors, and mortality of neonatal and late-onset dilated cardiomyopathy associated with cardiac neonatal lupus. Int J Cardiol. (2017) 248:263–9. 10.1016/j.ijcard.2017.07.100 28843719

[21] Doti PI Escoda O Cesar-Diaz S Palasti S Teixido I Sarquella-Brugada G. Congenital heart block related to maternal autoantibodies: descriptive analysis of a series of 18 cases from a single center. Clin Rheumatol. (2016) 35:351–6. 10.1007/s10067-016-3174-4 26791874

[22] Villain E Coastedoat-Chalumeau N Marijon E Boudjemline Y Piette JC Bonnet D. Presentation and prognosis of complete atrioventricular block in childhood, according to maternal antibody status. JACC. (2006) 48:1682–7. 10.1016/j.jacc.2006.07.034 17045907

[23] Santos-Pardo I Villuendas R Salvador-Corres I Martinez-Morillo M Olive A Bayes-Genis A. Anti-Ro/SSA antibodies and cardiac rhythm disturbances: Present and future perspectives. Int J Cardiol. (2015) 184:244–50. 10.1016/j.ijcard.2014.11.002 25725306

[24] Ambrosi A Sonesson S Wahren-Herlenius M. Molecular mechanisms of congenital heart block. Exp Cell Res. (2014) 325:2–9. 10.1016/j.yexcr.2014.01.003 24434353

[25] Karnabi E Boutjdir M. Role of calcium channels in congenital heart block. Scand J Immunol. (2010) 72:226–34. 10.1111/j.1365-3083.2010.02439.x 20696020 PMC2944231

[26] Boutjdir M Lazzerini PE Capecchi PL Laghi-Pasini F El-Sherif N. Potassium channel block and novel autoimmune-associated long QT syndrome. Card Electrophysiol Clin. (2016) 8:373–84. 10.1016/j.ccep.2016.02.002 27261828

[27] Udink TCF Breur JM Cohen MI Boramanand N Kapusta L Crosson JE Dilated cardiomyopathy in isolated congenital complete atrioventricular block: early and long-term risk in children. JACC. (2001) 37:1129–34. 10.1016/S0735-1097 11263619

[28] Aleksova A Carriere C Zecchin M Barbati G Vitrella G Di Lenarda A. New-onset left bundle branch block independently predicts long-term mortality in patients with idiopathic dilated cardiomyopathy: data from the Trieste Heart Muscle Disease Registry. Europace. (2014) 16:1450–9. 10.1093/europace/euu016 24550348

[29] Mahmod M Karamitsos TD Suttie JJ Myerson SG Neubauer S Francis JM. Prevalence of cardiomyopathy in asymptomatic patients with left bundle branch block referred for cardiovascular magnetic resonance imaging. Int J Cardiovasc Imaging. (2012) 28:1133–40. 10.1007/s10554-011-9931-1 21805313

